# Predictive Role of Prior Radiotherapy and Immunotherapy-Related Adverse Effects in Advanced NSCLC Patients Receiving Anti-PD-1/L1 Therapy

**DOI:** 10.3390/jcm10163719

**Published:** 2021-08-21

**Authors:** Jeong Uk Lim, Soo Han Kim, Hye Seon Kang, Sung Kyoung Kim, Ju Sang Kim, Jin Woo Kim, Seung Joon Kim, Chang Dong Yeo, Chang Min Choi

**Affiliations:** 1 Division of Pulmonary, Allergy and Critical Care Medicine, Department of Internal Medicine, Yeouido St. Mary’s Hospital, College of Medicine, The Catholic University of Korea, Seoul 07345, Korea; cracovian@catholic.ac.kr; 2 Asan Medical Center, Department of Pulmonary and Critical Care Medicine, University of Ulsan College of Medicine, Seoul 05505, Korea; kshyjt1004@gmail.com; 3 Division of Pulmonary and Critical Care Medicine, Department of Internal Medicine, Bucheon St. Mary’s Hospital, College of Medicine, The Catholic University of Korea, Seoul 14647, Korea; beyer_kr@catholic.ac.kr; 4 Division of Pulmonary, Critical Care and Sleep Medicine, Department of Internal Medicine, St. Vincent’s Hospital, College of Medicine, The Catholic University of Korea, Seoul 16247, Korea; kimskmd@gmail.com; 5 Division of Pulmonary, Allergy and Critical Care Medicine, Department of Internal Medicine, Incheon St. Mary’s Hospital, College of Medicine, The Catholic University of Korea, Seoul 21431, Korea; kimjusang@catholic.ac.kr; 6 Division of Pulmonary, Allergy and Critical Care Medicine, Department of Internal Medicine, Uijeongbu St. Mary’s Hospital, College of Medicine, The Catholic University of Korea, Seoul 11765, Korea; medkjw@catholic.ac.kr; 7 Division of Pulmonary and Critical Care Medicine, Department of Internal Medicine, Seoul St. Mary’s Hospital, College of Medicine, The Catholic University of Korea, Seoul 06591, Korea; cmcksj@catholic.ac.kr; 8 Cancer Research Institute, College of Medicine, The Catholic University of Korea, Seoul 06591, Korea; 9 Division of Pulmonary, Critical Care and Sleep Medicine, Department of Internal Medicine, Eunpyeong St. Mary’s Hospital, College of Medicine, The Catholic University of Korea, Seoul 03312, Korea; 10 Asan Medical Center, Department of Oncology, University of Ulsan College of Medicine, Seoul 05505, Korea

**Keywords:** immunotherapy, radiotherapy, adverse event, lung cancer

## Abstract

The present study evaluated the impact of prior radiotherapy (RT) on patients with advanced non-small cell lung cancer (NSCLC) receiving therapy with immune checkpoint inhibitors (ICIs) and further assessed the prognostic factors in patients receiving both RT and ICI. Patients diagnosed with NSCLC at the Catholic Medical Center and Asan Medical Center between January 2016 and October 2020 and who received immunotherapy were retrospectively reviewed. Among 240 patients, poor Eastern Cooperative Oncology Group (ECOG) score, high PD-L1 expression, and ICI-related adverse events (AE) were significantly associated with progression-free survival (PFS) (HR, 2.654; 95% CI, 1.484–4.749; *p* = 0.001; HR, 0.645; 95% CI, 0.449–0.926, *p* = 0.017; HR, 0.430; 95% CI, 0.229–0.808; *p* = 0.009, respectively). Among patients who received both RT and immunotherapy, poor ECOG status, squamous cell carcinoma, and ICI-related AE were significant factors associated with poor PFS (HR, 2.430; 95% CI, 1.464–4.034; *p* = 0.001; HR, 0.667; 95% CI, 0.455–0.978, *p* = 0.038; HR, 0.520; 95% CI, 0.284–0.953, *p* = 0.034, respectively). The present study showed that prior RT showed no significant independent association with primary outcomes in patients with advanced NSCLC receiving immunotherapy. In patients who received both RT and immunotherapy, clinical parameters, including ICI-related AEs, were independently predictive of PFS.

## 1. Introduction

Globally, lung cancer is one of the major causes of cancer-related deaths [1,2], and non-small cell lung cancer (NSCLC) accounts for 85% of all lung cancer cases [3]. Immune checkpoint inhibitors (ICIs) have become a main anti-cancer treatment modality in NSCLC patients, based on understanding the PD-1/PD-ligand 1 (PD-L1) pathway in the tumor-immune microenvironment [4,5,6]. Nivolumab, a PD-1 ICI antibody, significantly improved survival in patients previously treated for advanced NSCLC compared with docetaxel [7,8]. Pembrolizumab and atezolizumab have also shown favorable results [9,10].

Patients with locally advanced or metastatic NSCLC receive radiotherapy (RT) alone or in combination with chemotherapy. In addition to providing good local control of tumor growth, several studies have shown that radiation may have a strong immunomodulatory potential, making tumor cells more vulnerable to immune reactions [11]. Radiation induces DNA damage in tumor cells and increases reactive oxygen species (ROS); in turn, the immunogenicity of tumor cells becomes more activated, enabling an immune response to be mounted against the tumor cells [12,13]. Furthermore, it has been suggested that local RT can induce a decrease in the volume of untreated cancer lesions, and this notion of a systemic response due to localized RT is often termed the abscopal effect [14,15].

The PACIFIC trial investigated the role of adjuvant ICI after chemoradiotherapy in curable stage III NSCLC [16]. In addition to improved patient outcomes, the trial showed a slight increase in toxic effects in the durvalumab group compared with the placebo arm; however, the rates of severe immune-related adverse events, pneumonitis in particular, were not significantly different. In addition, patients showed lower disease recurrence when durvalumab was initiated within ≤2 weeks of the last radiation dose rather than being initiated >2 weeks after radiation. This may suggest that a short window between RT and the start of ICI may have affected clinical outcomes; however, it should also be considered that a selection bias may be present, in which patients with optimal conditions start sooner with consolidation treatment [17]. Several other studies have also evaluated the clinical impact of sequential or concurrent RT on patients with NSCLC undergoing immunotherapy. Shaverdian et al. showed that the median progression-free survival (PFS) was significantly longer in patients who received previous RT before pembrolizumab and that overall survival (OS) was also significantly longer in the same group than that in patients who did not receive prior RT [18]. In a multicenter, randomized phase II study (PEMBRO-RT), the objective response rate was higher in the experimental group that received RT prior to pembrolizumab than that in the control group that did not receive prior RT (36% vs. 18%, *p* = 0.07) [15]. A systematic review of 1736 patients treated with an ICI and stereotactic ablative RT (SABR) showed good local control rate (71%) and fair systemic control rate (41%) [19]. However, prior RT was not a predictive factor in a multicenter, retrospective cohort study in Japan [20].

Although various studies have shown that subsequent or concurrent RT and immunotherapy did not increase the risk of anti-PD-1-related toxicities [19,21,22], additional data on the combination treatment are necessary to better demonstrate the clinical impact and safety profile of the combination treatment, as the sample size of the populations varied and heterogeneity in terms of clinical characteristics was present.

The present study evaluated the impact of prior RT in patients with advanced NSCLC receiving ICI and further assessed potential prognostic factors in patients receiving both RT and ICI.

## 2. Methods

### 2.1. Patient Selection

Patients diagnosed with NSCLC at the Catholic Medical Center and Asan Medical Center between January 2016 and October 2020 and who received immunotherapy were included in this study. The seven university hospitals affiliated with the Catholic Medical Center are as follows: Yeouido St. Mary’s Hospital, Seoul St. Mary’s Hospital, Bucheon St. Mary’s Hospital, Incheon St. Mary’s Hospital, Eunpyeong St. Mary’s Hospital, St. Vincent Hospital, and Uijeongbu St. Mary’s Hospital.

The following data were retrospectively collected from electronic medical records: patient demographics, pathological characteristics, Eastern Cooperative Oncology Group (ECOG) score, accompanying driver mutations, immunotherapy regimen, previous history of RT, objective of RT, irradiated region and dose, previous chemotherapy history, and other parameters.

### 2.2. Outcomes

The follow-up duration was calculated from the date of first ICI infusion. OS was calculated from the first cycle of ICI infusion until the patient’s death or when lost to follow-up. Progression-free survival (PFS) was calculated from the first cycle of ICI infusion until the day of progression while on ICI or until the day of last ICI infusion in cases where patients did not show progression during ICI treatment. Disease progression was determined based on RECIST 1.1 criteria. Patients who underwent response evaluation were categorized as responders if they showed complete response, partial response, stable disease, or complete response during at least one point of response assessment, and if otherwise, they were categorized as non-responders [23].

### 2.3. Adverse Events

Adverse events (AEs) were evaluated according to the criteria of CTCAE 4.0. ICI-related AEs (ICI-AEs) were managed according to the recommended algorithms [24]. Corticosteroids were usually used to manage ICI-AEs. In severe ICI-AEs, the ICI dose was tapered, or in some cases, its use was discontinued.

### 2.4. Statistical Analysis

The Statistical Package for Social Sciences software (version 20.0; SPSS Inc., Chicago, IL, USA) was used to perform statistical analyses. Data of the continuous variables are shown as a mean or median with a range and were compared using two-sided t-tests or the Mann–Whitney U-test depending on the distribution status. The Chi-squared test was used for categorical variables. The OS and PFS of the patients were estimated using Kaplan–Meier survival curves, and the statistical difference between the groups was tested using the log-rank test. The Cox proportional hazard model was used to identify independent prognostic factors for OS and PFS. Variables that were statistically significant in the univariate analysis were entered into the multivariate analysis. Hazard ratios (HRs) and 95% confidence intervals (95% CIs) were estimated. Logistic regression analysis was used to determine the association between ICI-related adverse events. Statistical significance was set at *p* < 0.05.

### 2.5. Ethics Statement

The present study was approved by the Ethics Committees of Seoul St. Mary’s Hospital, Incheon St. Mary’s Hospital, Yeouido St. Mary’s Hospital, Bucheon St. Mary’s Hospital, Eunpyeong St. Mary’s Hospital, St. Vincent Hospital, and Uijeongbu St. Mary’s Hospital (XC19RIDI0110P). The Institutional Review Board of Asan Medical Center approved this study (approval no. 2020-004). The need for informed consent was waived by the Institutional Review Board.

## 3. Results

### 3.1. Clinical Characteristics of the Patients

A total of 240 patients who were administered ICI were evaluated. Among them, 57 patients did not receive prior RT and 183 patients received RT prior to initiation of ICI treatment. The mean age of the patients was 64.1 ± 9.1. Among all patients, 187 (77.9%) were male and 180 (75.6%) were ever smokers. The median OS was 24.3 months, and the median PFS was 5.4 months. Of all patients, 194 (88.6%) showed relatively good performance, with an ECOG score of 0–1. Regarding pathological subtypes of the patients, 121 (50.6%) patients had adenocarcinoma, 110 (46.0%) patients had squamous carcinoma, and 8 (3.3%) patients had other pathological types. While 45 (23.4%) patients had brain metastasis at the time of ICI initiation, 121 (50.6%) patients were treated with pembrolizumab and 118 (49.4%) patients were treated with nivolumab (Supplementary Table S1).

A group of patients that received prior RT was compared with a group that did not receive prior RT. The mean age of the RT group was lower than that of the no-RT group (62.8 vs. 68.2, *p* < 0.001). The proportion of men was higher in the RT group (83.6% vs. 59.6%, *p* < 0.001). Median OS was significantly longer in the RT group (27.1 months vs. 17.5 months, *p* = 0.005) (Supplementary Figure S1); however, median PFS was significantly shorter in the RT group (4.2 months vs. 7.8 months, *p* = 0.025) when compared with the no-RT group. The RT group showed a significantly higher percentage of squamous type tumors than the no-RT group (51.6% vs. 28.1%, *p* = 0.003). No statistically significant difference was observed in the proportion of epidermal growth factor receptor (EGFR) mutations and PD-L1 expression. The RT group showed a significantly lower percentage of brain metastases but tended to have more metastatic lesions at the time of ICI initiation. The number of previous chemotherapy cycles was higher in the RT group (1.92 vs. 1.47, *p* = 0.009). Moreover, the RT group showed a higher proportion of nivolumab as an ICI regimen (55.5% vs. 29.8%, *p* = 0.001) (Supplementary Table S1).

### 3.2. Comparison between ICI Responders and Non-Responders

Of all patients, 148 showed stable disease, partial response, or complete response during at least one point of the response assessment while 88 patients showed progressive disease at best. The responder group showed a significantly older mean age (65.1 vs. 61.2, *p* = 0.006) and a higher proportion of good performance patients (92.8% vs. 82.5%, *p* = 0.020). The non-responder group showed a higher proportion of patients with ≥4 metastatic lesions (19.3% vs. 9.5%, *p* = 0.034). The responder group showed a higher proportion of ICI-related AEs (16.9% vs. 5.7%, *p* = 0.012). The percentages of patients who received radiotherapy treatment prior to ICI initiation were 73.0% for the responder group and 80.7% for the non-responder group (*p* = 0.181). The target of radiotherapy (thorax vs. non-thorax), objective of radiotherapy (curative vs. non-curative), and interval between radiotherapy and immunotherapy initiation did not show significant difference between the groups (Table 1).

### 3.3. Association with PFS and OS in All Patients

PFS, age, ECOG, PD-L1 expression, number of metastatic lesions, immunotherapy-related AE, and prior RT showed statistically significant associations in the univariate analysis. Factors significant in the univariate analysis and sex were included in the multivariate analysis. Good ECOG score, high PD-L1 expression, and ICI-AE were significantly associated with longer PFS (HR: 2.654, 95% CI: 1.484–4.749, *p* = 0.001; HR: 0.645, 95% CI: 0.449–0.926, *p* = 0.017; HR: 0.430, 95% CI: 0.229–0.808, *p* = 0.009, respectively).

OS, age, number of metastatic lesions, and prior RT showed a statistically significant association in the univariate analysis. Factors significant in the univariate analysis and sex were included in the multivariate analysis. Only the number of metastatic lesions was significantly associated with OS (*p* = 0.016) (Table 2).

### 3.4. Association with PFS and OS in Patients who Underwent Prior RT

**Figure 1:**
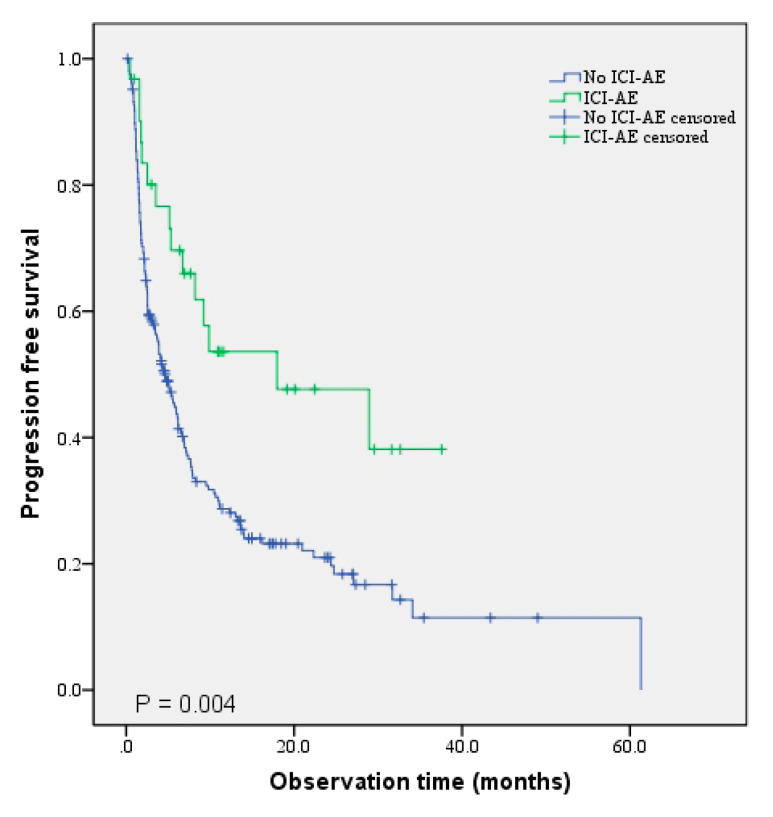
Comparison of PFS between the ICI-AE and without-ICI-AE groups.

Associations between PFS and OS were analyzed using the Cox regression hazard model in patients who received RT prior to ICI initiation. The univariate analyses for PFS, ECOG, pathological subtype, and presence of ICI-related AEs were significant. Factors significant in the univariate analysis, age, and sex were included in the multivariate analysis. Good ECOG score, pathological subtype (non-squamous vs. squamous), and ICI-related AE were significantly associated with longer PFS in the analysis (HR: 2.430, 95% CI: 1.464–4.034, *p* = 0.001; HR: 0.667, 95% CI: 0.455–0.978, *p* = 0.038; HR: 0.520, 95% CI: 0.284–0.953, *p* = 0.034, respectively). Figure 1 shows the Kaplan–Meier graph, which compares PFS between a group with ICI-AE and a group without ICI-AE. The ICI-AE group showed significantly longer PFS than the group without ICI-AE (*p* = 0.004) (Figure 1).

In the univariate analysis for OS, the number of metastatic lesions alone was significant. The multivariate analysis including sex, age, and factors that were significant in the univariate analysis also showed that only the number of metastatic lesions was significant (*p* = 0.023) (Table 3).

## 4. Discussion

The present study showed that RT prior to ICI initiation did not independently affect OS and PFS in patients with advanced NSCLC. However, the presence of immune-related AEs was significantly associated with PFS. This association was repeatedly observed in the group that received RT prior to ICI therapy.

Notably, ICI-AEs were independently associated with a better PFS in our study. Among the patients who received prior RT, 13.7% experienced ICI-related AEs. This percentage is lower than that reported by Lesueur et al. (59.6%) [21] and by Bang et al. (34.6%) [25]. It is possible that mild AEs of grade 1–2 could be underreported in our study. Although not shown in the results, the occurrence of ICI-AE did not demonstrate a significant difference between the group that received prior RT and the group that did not. Furthermore, among patients who received prior RT, the proportion of patients with ICI-AE did not show a significant difference between the patients with an interval between prior RT and the initiation of ICI-AE of shorter and longer than six months. The safety profile of concurrent or sequential RT in patients with NSCLCs under immunotherapy has been evaluated in several studies. In a study by Hubbeling et al., treatment with ICI and cranial RT was not associated with increased RT-related AEs in patients with advanced NSCLC with brain metastases [26], and a randomized clinical trial (PEMBRO-RT) showed that stereotactic body RT prior to pembrolizumab was well tolerated [15]. A multicenter, retrospective study in France also demonstrated that additional RT was not associated with increased risk of severe toxicities [21]. Another multicenter, retrospective study by Lesueur et al. also showed that PFS was significantly better in patients with ICI-AE than in those without ICI-AEs (*p* = 0.038) [21]. The addition of local radiotherapy in patients with oligometastatic or oligoprogressive disease while on immunotherapy was proven to be efficacious in several studies [27]. A retrospective analysis including NSCLC patients treated with stereotactic radiosurgery for brain metastases combined with checkpoint inhibitors showed that patients receiving RT concomitantly with immunotherapy had a longer OS (24.7 months) when compared with patients under both treatment but not concurrently (14.5 months) [28]. A single-arm phase 2 trial including 51 patients with oligometastatic NSCLC showed that pembrolizumab after local ablative therapy for oligometastatic NSCLC improved PFS (median PFS of 19.1 months) [29].

The association between ICI-AE and improved outcomes has been reported in several studies. Hwang et al. showed an association between the development of grade 2 or higher ICI-AEs and improved survival. The authors further discussed that patients showing response to the therapy are more likely to receive more cycles of ICI, which in turn increases the possibility of developing toxic or adverse effects [30].

In the Kaplan-Meier curve analysis, patients who received prior RT showed shorter PFS but longer OS to immunotherapy compared with those who did not. The opposing results of OS and PFS seemed unusual, but we assume that other factors such as tumor burden or age may have contributed to the results. The group who did not receive radiotherapy showed significantly older age when compared with the prior RT group, while the RT group showed significantly more patients who showed a high number of metastatic sites. However, in the multivariate analysis, prior RT did not affect OS or PFS in the present study. A meta-analysis including 20 clinical trials showed that combination therapy using immunotherapy and RT may improve OS, PFS, and tumor response rates without an increase in serious AEs in advanced NSCLC patients [22]. The study also suggested that the synergistic effect of combination treatment is more evident in patients undergoing stereotactic body RT or stereotactic radiosurgery. Shaveridian et al. showed that, in patients who enrolled in the KEYNOTE 001 study and were under treatment with pembrolizumab, those who received previous treatment with RT had longer PFS and OS, with a tolerable safety profile, than those who did not receive previous RT [18]. Another retrospective study also showed that combined RT and nivolumab showed superior 1-year OS and PFS compared with nivolumab monotherapy [31], and a study by Ratnayake et. al. showed similar results. RT prior to or concurrent with nivolumab for metastatic NSCLC is associated with improved PFS [32]. A phase II trial comparing neoadjuvant durvalumab monotherapy and durvalumab plus stereotactic radiotherapy in patients with early-stage NSCLC showed that the combination treatment was well tolerated and was associated with a high response rate [33]. Conversely, a multicenter, retrospective study conducted in Japan showed that prior RT was not a predictive factor [20].

The reason that prior RT showed no significant association with the outcomes in the present study may be due to the limitations owing to the retrospective study design. It is difficult to interpret whether prior RT has an immunomodulatory effect in patients. The median interval between the last radiation treatment and initiation of ICI was not short. Many of the patients had an interval longer than 6 months [21,34], and it is difficult to state that the immunomodulatory effect of prior RT remained. Furthermore, it is possible that patients with longer intervals are likely to have a relatively more stable clinical course. Thus, heterogeneity regarding the timing of radiotherapy and the disease burden of the patients should be controlled to show the effect of prior RT on ICI treatment more accurately. Prospective studies such as the PACIFIC-2 (NCT03519971) and PACIFIC-4 (NCTR03833154) are expected to show the clinical impact of the combination of immunotherapy and RT more accurately.

This study has several limitations. First, the purpose of radiotherapy was palliative in the majority of patients, and only a small proportion of patients received treatment for curative purposes. The group that received RT had a higher number of metastatic sites, and tumor burdens at the time of radiotherapy could have affected the analysis results. Second, because of the retrospective study design, selection bias may have affected the study results. Lastly, the percentage of irAEs was relatively low when compared with other studies [35,36]. This was due to the limitation in number of AEs checked; relatively common AEs such as hematologic abnormality, fatigue, and nausea were not checked.

## 5. Conclusions

The present study showed that prior RT has no significant association with primary outcomes in patients with advanced NSCLC receiving RT. In patients who receive both RT and immunotherapy, clinical parameters, including ICI-related AEs, were independently predictive of PFS. Future studies that consider disease burden, purpose and site of RT, and interval between prior RT and immunotherapy are necessary to more accurately demonstrate the synergistic effect of prior RT and immunotherapy.

## Figures and Tables

**Table 1:** Comparison of responders and non-responders among overall patients.

Parameters	Responders (*n* = 148)	Non-Responders (*n* = 88)	*p*-Value
Age, mean	65.1 ± 8.6	61.2 ± 9.5	0.006
OS (IQR) (months)	24.7 (15.7–30.7)	19.8 (11.4–31.6)	0.450
Sex			0.063
Male	109 (73.6)	74 (84.1)	
Female	39 (26.4)	14 (15.9)	
Smoking history			0.196
Ever	108 (73.0)	70 (80.5)	
Never	40 (27.0)	17 (19.5)	
ECOG			0.020
0–1	128 (92.8)	66 (82.5)	
2–4	10 (7.2)	14 (17.5)	
Pathology			0.879
Adenocarcinoma	75 (50.7)	46 (52.9)	
Squamous	69 (46.6)	38 (43.7)	
Others	4 (2.7)	3 (3.4)	
EGFR mutation	12/98 (12.2)	9/62 (14.5)	0.679
PD-L1 TPS (22C3)			0.177
<1%	7 (4.7)	7 (8.0)	
1-49%	45 (30.4)	28 (31.8)	
≥50%	76 (51.4)	37 (42.0)	
N/A	20 (13.5)	16 (18.2)	
Brain metastasis	31 (20.9)	23 (26.1)	0.359
No. of metastasis at the time of immunotherapy initiation			0.034
0	36 (24.3)	12 (13.6)	
1	43 (29.1)	18 (20.5)	
2	34 (23.0)	28 (31.8)	
3	21 (14.2)	13 (14.8)	
≥4	14 (9.5)	17 (19.3)	
No. of previous CTx lines			0.257
0	3 (2.0)	4 (4.5)	
1	76 (51.4)	41 (46.6)	
2	35 (23.6)	22 (25.0)	
3	17 (11.5)	6 (6.8)	
≥4	17 (11.5)	15 (17.1)	
Prior radiotherapy	108 (73.0)	71 (80.7)	0.181
Thorax RT	77 (71.3)	48 (67.6)	0.599
Non-thorax RT	31 (28.7)	23 (32.4)	
Curative RT	57 (52.8)	29 (40.8)	0.118
Non-curative RT	51 (47.2)	42 (59.2)	
Interval between RT and immunotherapy initiation			0.381
<60 days	20 (18.5)	17 (23.9)	
≥60 days	88 (81.5)	54 (76.1)	
Immunotherapy			0.313
Pembrolizumab	79 (53.4)	41 (46.6)	
Nivolumab	69 (46.6)	47 (53.4)	
ICI-AE	25 (16.9)	5 (5.7)	0.012

Abbreviations: OS; overall survival, IQR; interquartile range, ECOG; Eastern Cooperative Oncology Group, EGFR; epidermal growth factor receptor tyrosine kinase, PD-L1; programmed death-ligand 1, CTx; chemotherapy, ICI-AE; immune checkpoint inhibitor-related adverse events, TPS; tumor proportion score.

**Table 2:** Evaluation of association of clinical parameters with PFS and OS.

Parameters		PFS			OS		
		Univariate	Multivariate		Univariate	Multivariate	
		*p*-Value	HR (95%CI)	*p*-Value	*p*-Value	HR (95%CI)	*p*-Value
Sex	Male (Ref)						
	Female	0.150	0.834 (0.542–1.283)	0.409	0.739	1.158 (0.708–1.894)	0.559
Age	Year	0.012	0.984 (0.961–1.007)	0.165	0.028	1.008 (0.981–1.037)	0.556
ECOG	0–1 (Ref)		1		0.409		
	2–4	0.002	2.654 (1.484–4.749)	0.001			
Smoking history	Never smoker (Ref)				0.608		
	Ever smoker	0.211					
Pathology	Nonsquamous (Ref)						
	Squamous	0.279			0.153		
EGFR	Wild-type (Ref)						
	Mutation	0.473			0.575		
PD-L1 (22C3)	<50% (Ref)		1				
	≥50%	0.029	0.645 (0.449–0.926)	0.017	0.098		
CNS metastasis	No (Ref)						
	Yes	0.132			0.650		
No. of metastasis	0 (Ref)	0.012	1	0.174	<0.001	1	0.016
	1		1.306 (0.749–2.278)	0.346		0.588 (0.309–1.118)	0.105
	2 and more		1.615 (0.964–2.706)	0.068		0.400 (0.213–0.751)	0.004
Immunotherapy related AE	No (Ref)						
	Yes	0.005	0.430 (0.229–0.808)	0.009	0.663		
Prior radiotherapy	No (Ref)						
	Yes	0.027	1.027 (0.642–1.644)	0.912	0.006	0.838 (0.476–1.474)	0.539

Abbreviations: PFS; progression free survival, OS; overall survival, ECOG; Eastern Cooperative Oncology Group, EGFR; epidermal growth factor receptor tyrosine kinase, PD-L1; programmed death-ligand 1, AE; adverse events.

**Table 3:** Cox regression for PFS and OS in patients who received prior radiotherapy.

Parameters		PFS				OS			
		Univariate		Multivariate		Univariate		Multivariate	
		HR (95%CI)	*p*-Value	HR (95%CI)	*p*-Value	HR (95%CI)	*p*-Value	HR (95%CI)	*p*-Value
Sex	Male (Ref)								
	Female	0.764 (0.478–1.219)	0.259	0.790 (0.475–1.314)	0.364	0.883 (0.464–1.682)	0.706	1.039	0.910
Age	Year	0.986 (0.967–1.006)	0.178	0.988 (0.966–1.012)	0.328	1.028 (0.996–1.061)	0.090	1.022 (0.989–1.056)	0.195
ECOG	0-1 (Ref)								
	2-4	2.305 (1.402–3.789)	0.001	2.430 (1.464–4.034)	0.001	0.230 (0.030–1.749)	0.156		
Smoking history	Never smoker (Ref)								
	Ever smoker	1.404 (0.903–2.184)	0.132			1.316 (0.716–2.416)	0.377		
Pathology	Nonsquamous (Ref)								
	Squamous	0.707 (0.501–0.997)	0.048	0.667 (0.455–0.978)	0.038	0.719 (0.420–1.230)	0.228		
EGFR	Wild-type (Ref)								
	Mutation	1.087 (0.596–1.984)	0.785			0.463 (0.156–1.371)	0.164		
PD-L1 (22C3)	<50% (Ref)								
	≥50%	0.720 (0.488–1.062)	0.098			0.575 (0.328–1.010)	0.054		
CNS metastasis	No (Ref)								
	Yes	1.156 (0.789–1.694)	0.457			1 (0.529–1.892)	1.000		
No. of metastasis	0								
	1	1.047 (0.541–2.026)	0.892			0.411 (0.181–0.931)	0.033	0.431 (0.188–0.987)	0.030
	≥2	1.565 (0.854–2.868)	0.147			0.282 (0.124–0.641)	0.003	0.305 (0.131–0.711)	0.005
RT-ICI interval	Day	1.000 (0.999–1.000)	0.281			1.000 (1.000–1.001)	0.471		
ICI related AE	No (Ref)								
	Yes	0.474 (0.267–0.841)	0.011	0.520 (0.284–0.953)	0.034	1.397 (0.710–2.748)	0.333		
RT intention	Curative (Ref)								
	Else	1.393 (0.989–1.962)	0.058			1.350 (0.792–2.299)	0.270		
RT target	Thorax (Ref)								
	Non-thorax	1.062 (0.734–1.538)	0.749			0.948 (0.497–1.806)	0.871		
RT pneumonitis	No (Ref)								
	Yes	0.850 (0.596–1.212)	0.369			0.783 (0.451–1.359)	0.385		
ICI pneumonitis	No (Ref)								
	Yes	0.445 (0.182–1.089)	0.076			1.713 (0.612–4.797)	0.306		

Abbreviations: PFS; progression free survival, OS; overall survival, ECOG; Eastern Cooperative Oncology Group, EGFR; epidermal growth factor receptor tyrosine kinase, PD-L1; programmed death-ligand 1, CNS; central nervous system, AE; adverse events, RT; radiotherapy, ICI; immune checkpoint inhibitor.

## Data Availability

The data presented in this study are available from the corresponding author upon request.

## Supplementary Materials

The following are available online at https://www.mdpi.com/article/10.3390/jcm10163719/s1. Figure S1: Comparison of OS between patients who received prior radiotherapy and those who did not, Table S1: Comparison of clinical characteristics between patients under radiotherapy and those who did not (*n* = 240).
