# Supplementary material for: Predictive Role of Prior Radiotherapy and Immunotherapy-Related Adverse Effects in Advanced NSCLC Patients Receiving Anti-PD-1/L1 Therapy
Source: J Clin Med. 2021 Aug 21;10(16):3719. doi: 10.3390/jcm10163719 (PMC8397093; doi:10.3390/jcm10163719)
Supplement: Supplementary file 1 [file jcm-10-03719-s001.zip › jcm-1304469-supplementary.pdf]

Journal of
Clinical Medicine

Supplementary Materials

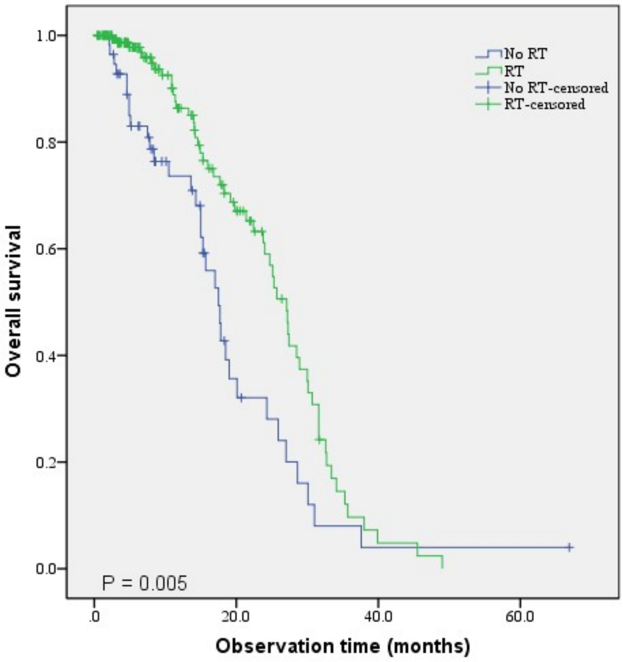

Supplementary Figure S1. Comparison of OS between patients who received prior radiotherapy and those who did not.

Supplementary Table S1. Comparison of clinical characteristics between patients under radiotherapy and those who did not (*n* = 240)

Parameters	Overall (n=240)	No RT (n=57)	RT (n=183)	P-value
Age, mean	64.1±9.1	68.2±9.1	62.8±8.8	<0.001
Sex				<0.001
Male	187 (77.9)	34 (59.6)	153 (83.6)	
Female	53 (22.1)	23 (40.4)	30 (16.4)	
OS, median, IQR (months)	24.3 (16.0-31.6)	17.5 (10.5-25.9)	27.1 (16.0-31.6)	0.005
PFS, median, IQR (months)	5.4 (1.7-22.3)	7.8 (3.9-22.3)	4.2 (1.6-18.0)	0.025
Smoking history				0.071
Never	58 (24.4)	19 (33.3)	39 (21.5)	
Ever	180 (75.6)	38 (66.7)	142 (78.5)	
ECOG				0.225
0-1	194 (88.6)	53 (93.0)	141 (87.0)	
2-4	25 (11.4)	4 (7.0)	21 (13.0)	
Pathology				0.003
Adenocarcinoma	121 (50.6)	40 (70.2)	81 (44.5)	
Squamous	110 (46.0)	16 (28.1)	94 (51.6)	
Others	8 (3.3)	1 (1.8)	7 (3.8)	
EGFR mutation	21/161 (13.0)	5/57 (8.8)	16/104 (15.4)	0.233
PD-L1 TPS (22C3)				0.205
<1%	15 (7.4)	2 (3.6)	13 (8.9)	
1-49%	73 (36.1)	16 (28.5)	57 (39.0)	
≥50%	114 (56.4)	38 (67.9)	76 (52.1)	
Brain metastasis	56 (23.4)	7 (12.3)	49 (6.9)	0.023
No. of metastasis at the time of immunotherapy initiation				<0.001
0	48 (20.0)	27 (47.4)	21 (11.5)	

1	61 (25.4)	13 (22.8)	48 (26.2)	
2	62 (25.8)	10 (17.5)	52 (28.4)	
3	35 (14.6)	3 (5.3)	32 (17.5)	
4 and more	33 (13.8)	4 (7.0)	29 (15.8)	
No. of previous CTx lines	1.81±1.13	1.47±1.10	1.92±1.12	0.009
Immunotherapy				0.001
Pembrolizumab	121 (50.6)	40 (70.2)	81 (44.5)	
Nivolumab	118 (49.4)	17 (29.8)	101 (55.5)	

Abbreviations: OS; overall survival, PFS; progression free survival, IQR; interquartile range, ECOG; Eastern Cooperative Oncology Group, EGFR; epidermal growth factor receptor tyrosine kinase, PD-L1; programmed death-ligand 1, CTx; chemotherapy, ICI-AE; immune checkpoint inhibitor-related adverse events, RT; radiotherapy, TPS; tumor proportion score
